# Spectroscopic evaluation of sesame and mustard oils treated with *Murchana* method

**DOI:** 10.1007/s10103-024-04050-x

**Published:** 2024-04-11

**Authors:** S Deekshitha, Kausalya Neelavara Makkithaya, Sharmila Sajankila Nadumane, Gazala Hussain, Sib Sankar Mal, Babitha K. Sundara, Padmini Pai, Nirmal Mazumder

**Affiliations:** 1 Department of Rasashastra and Bhaishajya Kalpana, Sri Dharmasthala Manjunatheshwara College of Ayurveda & Hospital, Hassan, 573201 India; 2 Department of Biophysics, Manipal School of Life Sciences, Manipal Academy of Higher Education, Manipal, 576104 India; 3 Materials and Catalysis Lab, Department of Chemistry, National Institute of Technology Karnataka, Surathkal, Karnataka 575025 India

**Keywords:** Vegetable oils, FTIR spectroscopy, UV-Vis absorbance, Fluorescence, *Murchana* process

## Abstract

In recent years, there has been a growing interest in traditional medicinal practices such as Ayurveda, which emphasizes the use of natural ingredients for various therapeutic purposes. Vegetable oils are an integral part of our diet and have several applications in the cosmetics and healthcare industries. These oils have also been prescribed in ancient Ayurveda texts to treat various health problems. Ayurveda prescribes a processing technique called ‘Murchana’ to improve the therapeutic nature of the oils. Spectroscopic techniques have been used for quality assessment in many fields. High sensitivity and a low detection rate make spectroscopy a formidable analytical technique. This study focusses on the spectroscopic analysis of sesame and mustard oils prepared using the ayurvedic processing method ‘Murchana’. Spectroscopic analysis techniques including UV-Vis absorbance spectroscopy, fluorescence spectroscopy, and FTIR spectroscopy were employed to study the oils. Origin software was used to plot graphs of the spectra. The results indicated that the *murchana* process may reduce the components of the oil responsible for its oxidation, thereby increasing the shelf life of the oils. However, further investigations, including other spectroscopy and chromatography techniques, will prove beneficial in ascertaining the effects of the *murchana* process on vegetable oils. The study’s findings also suggest that spectroscopic techniques can be used to supplement chemical techniques to investigate the characteristics of vegetable oils.

**Supplementary Information:**

The online version contains supplementary material available at 10.1007/s10103-024-04050-x.

## Introduction

Vegetable oils are oils extracted from plant sources such as seeds, fruits and their parts, or even whole plants in some cases. They have a plethora of industrial and household applications. Chemically, oils contain several compounds, such as triacylglycerol, free fatty acids, phosphatides, and chlorophylls [1].

The quality and authenticity of edible oils have garnered considerable attention due to their crucial role in human nutrition and health. Among various methods employed for oil processing, the traditional techniques hold significant cultural and historical relevance in many regions worldwide.

Ayurveda, a holistic health care system, advocates the usage of several medicated oils on the body to obtain several health benefits and for the treatment of specific conditions of the body [2–6]. Most oils prescribed by ayurveda are used for external applications [7]. In Ayurveda, oils are utilized for their therapeutic properties, derived from both animal and plant sources. Vegetable oils such as Mustard oil (*Sarshapa taila*) and Sesame oil (*Tila taila*) are extracted from various plant sources and widely employed as cooking oils [8]. However, before their incorporation into Ayurvedic treatments, these vegetable oils undergo a specialized process known as ‘*Murchana*’. This traditional practice aims to enhance the therapeutic efficacy of the taila while eliminating any signs of rancidity (Aama dosha), thereby extending its shelf life [9]. The treated tailas serve as a fundamental component for formulating various medicinal oils, as prescribed in Ayurvedic texts, to address a wide range of health conditions and disorders. *Murchana* is one of the first processing techniques or *samskara* followed in ayurveda oil preparation to remove the foul odour or *durgandha* of the oil and *aama dosha*. *Samskara* is a process of transformation of the natural qualities of a food substance or medicinal formulation by treatment through water, heating, and other processes such as churning, cleaning, and flavouring. *Samskara* aids in making the treated substance homogenous and thus compatible with the body depending on the composition of the body, or the *prakriti*. This also improves the therapeutic value of medicinal substances [10]. *Murchana samskara* was first described in detail in the ayurvedic classic Bhashajya Ratnavali (Chap. 5: Verses 1286-87). It is indicated that if a medicated oil is prepared without *Murchana*, the oil may be devoid of standard qualities, and thus hamper the beneficial effects of the oil. Furthermore, such medicated oils tend to become rancid, thus not giving the expected results from the medication [11]. The *Murchana* process also increases the saponification of the oil. As a result, the oil is able to imbibe the active properties of the drugs it is often combined with, increasing its potency. A lower acid value indicates the presence of free fatty acids. In comparison, a higher saponification value indicates the presence of more small chain fatty acids, and a reduced iodine value indicates the higher stability of the oil [12]. Processes such as *Murchana* also aid in making the medicine suitable to the *prakriti* of an individual, thus formulating a personised medicine [13]. *Murchana* process of sesame oil and mustard oil is described in the ayurveda classics such as Bhashajya Ratnavali and Sharangadhara Samhitha by Kashirams Gudarthadipika [14].

The *Murchana* process of sesame oil involves heating medicinal plants such as Manjistha, Haridra, Musta, Nalika, Amalaki, Vibhitaki, Haritaki, Suchipushpa, and Vata in the form of smooth paste with oil and water in the ratio of 1:4:16 on medium flame at a temperature of 90–940 C until the appearance of foam, evaporation of water content (assessed by putting the paste of the herbs on fire; if crackling sound ceases it infers the absence of moisture content), appreciation of colour, odour and taste of the drugs used. In case of mustard oil, the medicinal plants such as Bilva, Dadima, Nagakeshara, Krishna jeeraka, Ushira. The oil is then filtered, and this *murchitha* oil is used for other medicinal purposes [15, 16].

Processes such as *Taila Murchana*, prescribed by ayurveda are performed to enhance the medicinal capacity of the oils used in ayurvedic treatments. The current study aims to assess the changes occurring in the vegetable oils post the *Murchana* process. Vegetable oils have generally been analysed using various chemical and chromatography approaches. The changes in oil properties after being treated by the *Murchana* process was ascertained by physiochemical evaluations [17–19]. The pigments, especially chlorophyll plays an important role in assessing the quality of oils [20]. Lately, there has been an increasing demand for the application of instrumental analytical approaches for oil analyses and to replace traditional analytical methods. High sensitivity, low detection limits and increased speed of analysis are some of the advantages of instrumental analysis techniques such as spectroscopy. Spectroscopy techniques have found application in both qualitative and quantitative analyses in industries such as the chemical and food industries, electronic and metallurgy. Quality estimation of oils can be done through methods such as Fourier Transform Infrared (FTIR) spectroscopy, UV- VIS spectroscopy [21] and fluorescence spectroscopy [22] and High-Performance Liquid Chromatography (HPLC) due to the distinct spectra of each oil in their pure form, which is used as a marker to detect any changes or adulteration in the oils [23]. The choice of spectral technique depends on the specific goals of the analysis, the properties of the oil sample, and the required sensitivity and specificity of the analysis. Lasers play a pivotal role in enhancing spectroscopic techniques for the analysis of oil samples. Their unique properties, such as coherence, monochromaticity, and high intensity, enable precise and sensitive measurements. These lasers employed in spectroscopic techniques such as fluorescence spectroscopy offer rapid elemental analysis of oil samples by vaporizing small amounts of the sample and analysing the emitted light. The density of the oil sample can influence the absorption, scattering, and transmission of laser energy through the sample. Higher-density oil samples tend to absorb and scatter more laser energy compared to lower-density samples. This increased absorption and scattering can result in a decrease in the energy of the laser beam as it penetrates deeper into the sample. Therefore, understanding the density-dependent effects on laser energy is essential for optimizing spectroscopic techniques for the analysis of oil samples across a range of densities. Often, a combination of multiple techniques may be employed to obtain comprehensive information about the composition and quality of oil samples. Spectroscopic techniques such as absorbance spectroscopy, fluorescence spectroscopy, and Fourier-transform infrared (FTIR) spectroscopy are widely available and applicable to a diverse range of problems. The current study aims to demonstrate the applicability of spectroscopic methods such as UV- VIS spectroscopy, fluorescence spectroscopy and Fourier Transform Infrared (FTIR) spectroscopy as methods of analysis to evaluate the quality of plain mustard and sesame oils subject to the *murchana* process and that of the non-processed mustard and sesame oils.

In environmental science, absorbance spectroscopy is used for monitoring water quality parameters such as dissolved organic matter and pollutants. Fluorescence spectroscopy finds applications in studying environmental pollutants, analyzing biological samples, and monitoring chemical reactions. FTIR spectroscopy is extensively employed in pharmaceuticals for drug analysis, polymer science for material characterization, and forensic science for trace evidence analysis. Moreover, these techniques are utilized in food science for quality control, in medical diagnostics for disease detection, and in materials science for structural analysis. Their availability in various research and industrial settings makes them versatile tools for addressing a wide array of scientific and technological challenges across different disciplines [24].

## Materials and methods

*Murchitha* sesame oil and *murchitha* mustard oil were obtained from SDM College of Ayurveda and Hospital, Hassan, Karnataka, India. Plain sesame and mustard oil were collected from the market and used as control. These oils were obtained from the seeds on the plant and were processed through the cold pressing technique which is known to preserve the quality and natural properties of the oil. All oils have been stored at room temperature and light conditions (light and dark) until use. 200µL of samples each were loaded into flat bottom 96 - well plate for UV-Vis absorption and fluorescence spectroscopy. The samples were used for spectroscopic analysis at room temperature. The samples were opened on the same day, and the readings were taken periodically.

The oils were analysed by different spectroscopic methods in this study. The UV-Vis absorption spectra acquisition was carried out using Varioskan LUX Multimode Microplate Reader (Thermo Scientific, India) and the software used was SkanltTM soft-ware 2.4.3 RE, over the spectral range of ultraviolet and visible range, 280 nm to 750 nm, with a step size of 2 nm, bandwidth 5 nm, and measurement duration of 100ms. The samples were loaded (~ 200µL) in triplicates into a flat bottom 96 - well plate for acquisition of absorption spectra. The Varioskan microplate reader by Thermo Fisher Scientific offers several advantages, including its versatility in performing a wide range of absorbance, fluorescence, and luminescence assays, its modular design allowing for customization to meet specific experimental needs, and its high-throughput capabilities for simultaneous analysis of multiple samples. However, potential drawbacks include the initial high cost of purchase and optional accessories, the need for regular maintenance and calibration, and possible compatibility issues with certain assay protocols or reagents. Fluorescence spectra of the samples were acquired at 300 –420 nm excitation wavelengths and the emission were measured with an increment of 30 nm from the ex-citation wavelength to 750 nm, with a step size of 2 nm. SkanItTM Software 2.4.3 RE was used for the spectral acquisition with a measurement time of 100ms [25]. FTIR spectra of the samples were recorded using FTIR Spectrometer (Alpha II, Bruker, USA) equipped with attenuated total reflectance (ATR). The oil samples in this study (~ 50 µL) were loaded into the ATR, and spectra were acquired in the range of 400–4000 cm^− 1^ to analyse chemical composition of the oils. The samples’ UV – Visible absorption spectra [26], fluorescence [22] and FTIR spectra were recorded, after which the oils were stored for three months at room temperature. The FTIR Alpha II spectrometer by Bruker, USA, offers several advantages, including its high sensitivity and accuracy in Fourier-transform infrared spectroscopy analysis, its user-friendly interface facilitating easy operation and data interpretation, and its robust design ensuring reliable performance. However, potential drawbacks may include the relatively high initial cost of purchase and maintenance, as well as the need for specialized training to fully utilize its advanced features. After the duration, the oils’ UV – Visible absorption spectra, fluorescence and FTIR spectra was recorded again. The graphs of the data were extracted into excel sheets, and plotted using OriginPro software (version 2022b) (OriginLab Corporation, USA).

## Results and discussion

### UV – vis absorption spectroscopy

**Fig. 1:**
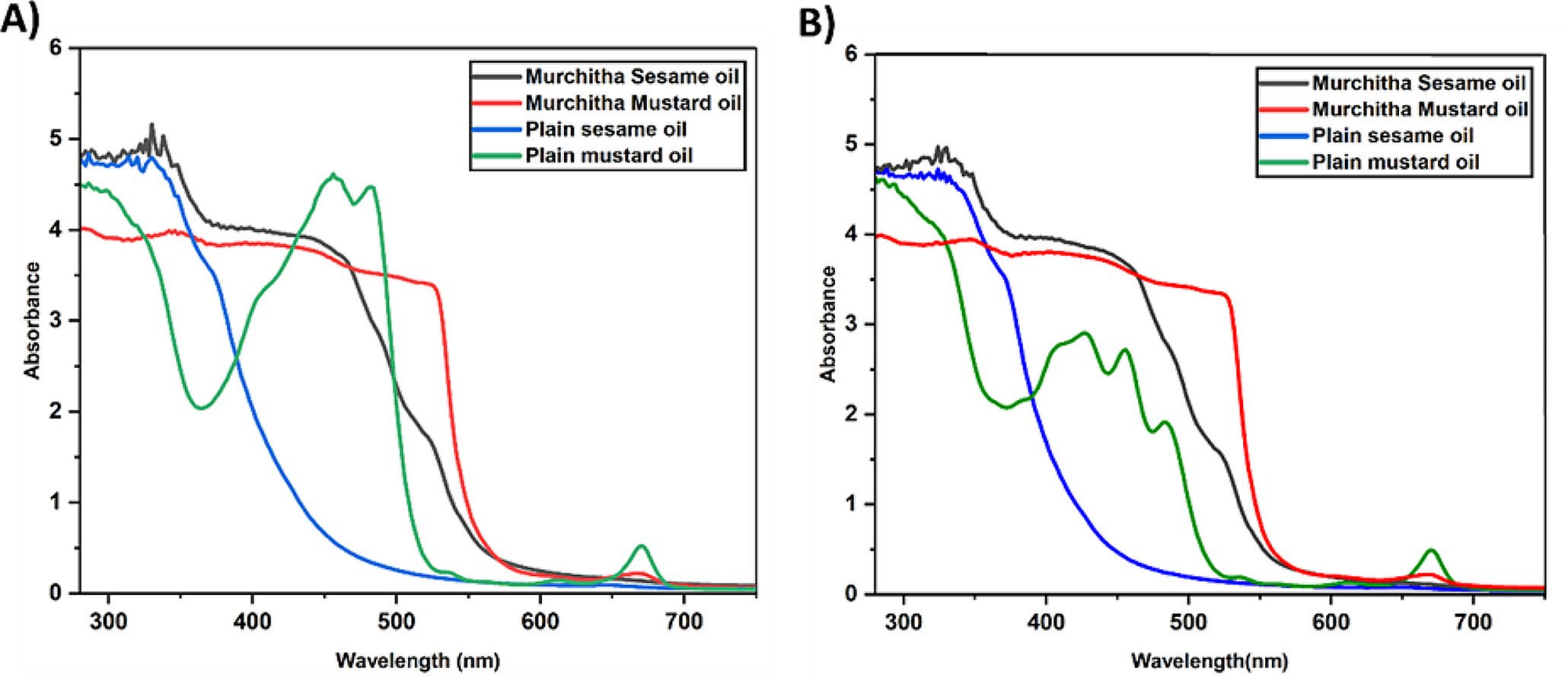
Absorbance spectra of the oil samples **A** Before storage and **B** 3 months after storage

UV-Visible absorption spectroscopy is employed to determine the absorption spectra of the various oil samples over three months. UV-Vis absorbance spectroscopy offers rapid and straightforward analysis of oils, providing valuable insights into their chemical composition and quality. This technique allows for the determination of key parameters such as the presence of impurities, oxidation levels, and the concentration of specific compounds within the oil sample. Additionally, UV-Vis spectroscopy is relatively cost-effective and widely accessible, making it suitable for the analysis of oil samples in the study. The different functional groups of chromophores and auxochromes in the vegetable oils contribute to the absorption spectrum of the oil. The absorption spectra of the *murchitha* sesame oil, *murchitha* mustard oil, and the corresponding plain or *amurchitha* oils acquired is depicted in Fig. 1. Among the samples investigated, it is observed that the *murchitha* sesame oil exhibited the maximum absorption at 330 nm with absorbance value 5.159. *Murchitha* mustard oil showed higher absorption levels at the initial stages of the study, a decline in the absorbance spectra is observed from 525 nm. The above observations are inferred from the graph before storage. After storage, *murchitha* sesame oil still had the highest absorbance of 4.964 at a wavelength 330 nm, and *murchitha* mustard oil absorbance peak also showed a trend similar to its absorbance peak before storage, with the lower inclination of peak beginning at 525 nm, with an absorbance value of 3.314 nm. The absorbance spectra of plain mustard oil show a drastic change before and after storage, from 4.585 absorbance value at 456 nm to 2.701 at the same wavelength. However, the absorption spectra of plain sesame oil have shown no significant change in the absorption levels before and after storage. The data in Fig. 1 shows significant peaks at 325 nm, indicating the presence of compounds such as tocopherols and phenolic compounds. Two distinctive peaks are observed in the absorption spectra of plain mustard oil, in the region between 400 and 500 nm, indicating the presence of carotenoids, a class of pigments present in oils, which impart a yellow tinge [27]. The absorption spectra indicate a strong presence of carotenoids in mustard oils. However, over a period of time, a dramatic drop in the absorption peak of the mustard oil was noticed due to photooxidative degradation of the carotenoids. Compared to plain oils obtained from the market, the oils treated through the *murchana* process oxidized more slowly. *Amurchitha* oils exhibit this peak at around 670 nm, indicating a high chlorophyll content. Chlorophyll is a critical compound in plants for metabolism. Oil extraction removes most of the chlorophyll. The trace remnants of the pigment impart a greenish tinge to the oils. This pigment is known to promote the oxidation of vegetable oils and contribute to the rancid smell and off-flavour of the oils [28, 29]. The data represented in Fig. 1 shows that mustard oils have the maximum chlorophyll content compared to sesame oil. Furthermore, from the figure, we can also see that the absorption peak for chlorophyll is lower in the case of *murchitha* mustard oil compared to the *amurchitha* mustard oil, thereby indicating the role of the *murchana* process in the reduction of the chlorophyll content in oils. This may be corresponding to a lower peroxide value in the *murchitha* taila. We can also observe a drastic decline in the absorption spectra of plain mustard oil over the period of storage, thus indicating a change in the quality of the oil. Plain sesame oil, however, shows stable behaviour before and after storage. This relative stability of the plain sesame oil can be attributed to the presence of gamma-tocopherol, lignans, and antioxidants in the oil [30]. The reduced chlorophyll content in the *murchitha* oils indicate a possible delay in the oxidation of the oils which is indicated as a stark decrease in the iodine value, peroxide value and acid value of the *murchitha* oils, in comparison to *amurchitha* oils [31–33]. These results indicate the stability of the vegetable oils during the storage period. The results also indicate the applicability of UV-Vis spectroscopy technique to assess the quality of vegetable oils. Despite these findings, one of the limitations of UV-Vis absorbance spectroscopy for oil analysis is its inability to provide detailed structural information about complex oil matrices. While UV-Vis spectroscopy can detect the presence of certain functional groups or chromophores such as chlorophyll within the oil molecules, it may not offer sufficient specificity for distinguishing between closely related compounds or detecting trace contaminants, thus necessitating the analysis of the oil samples using other supplementary techniques.

### Fluorescence spectroscopy

The intensity of fluorophores is assessed using fluorescence spectroscopy.

**Fig. 2:**
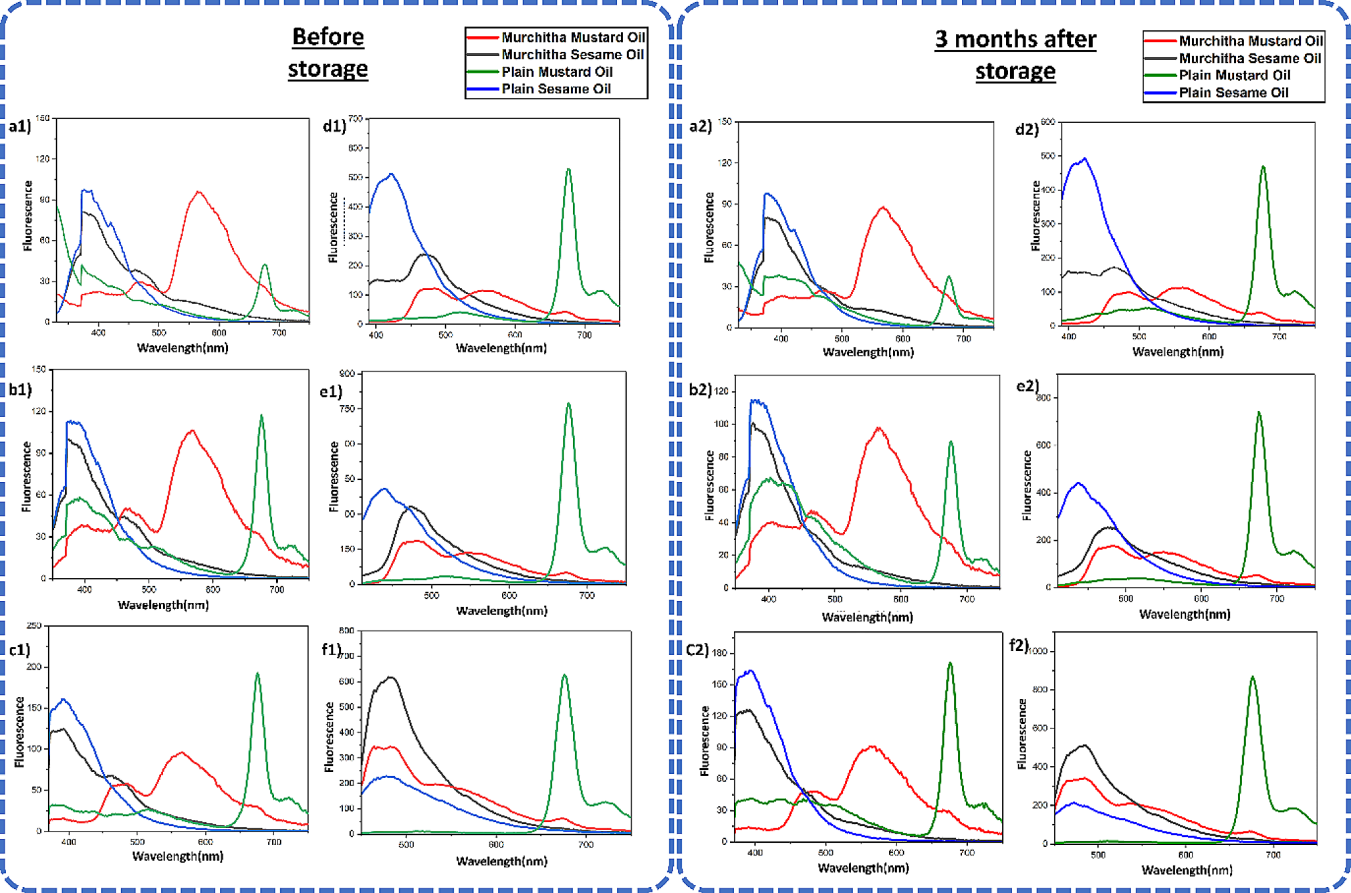
Fluorescence spectra **a1**, **a2** represents the fluorescence spectra of oils at 300 nm excitation **b1**, **b2** represents the fluorescence spectra of oils at 320 nm excitation **c1**, **c2** represents the fluorescence spectra of oils at 340 nm excitation **d1**, **d2** represents the fluorescence spectra of oils at 360 nm excitation **e1**, **e2** represents the fluorescence spectra of oils at 380 nm excitation **f1**, **f2** represents the fluorescence spectra of oils at 420 nm excitation

**Fig. 3:**
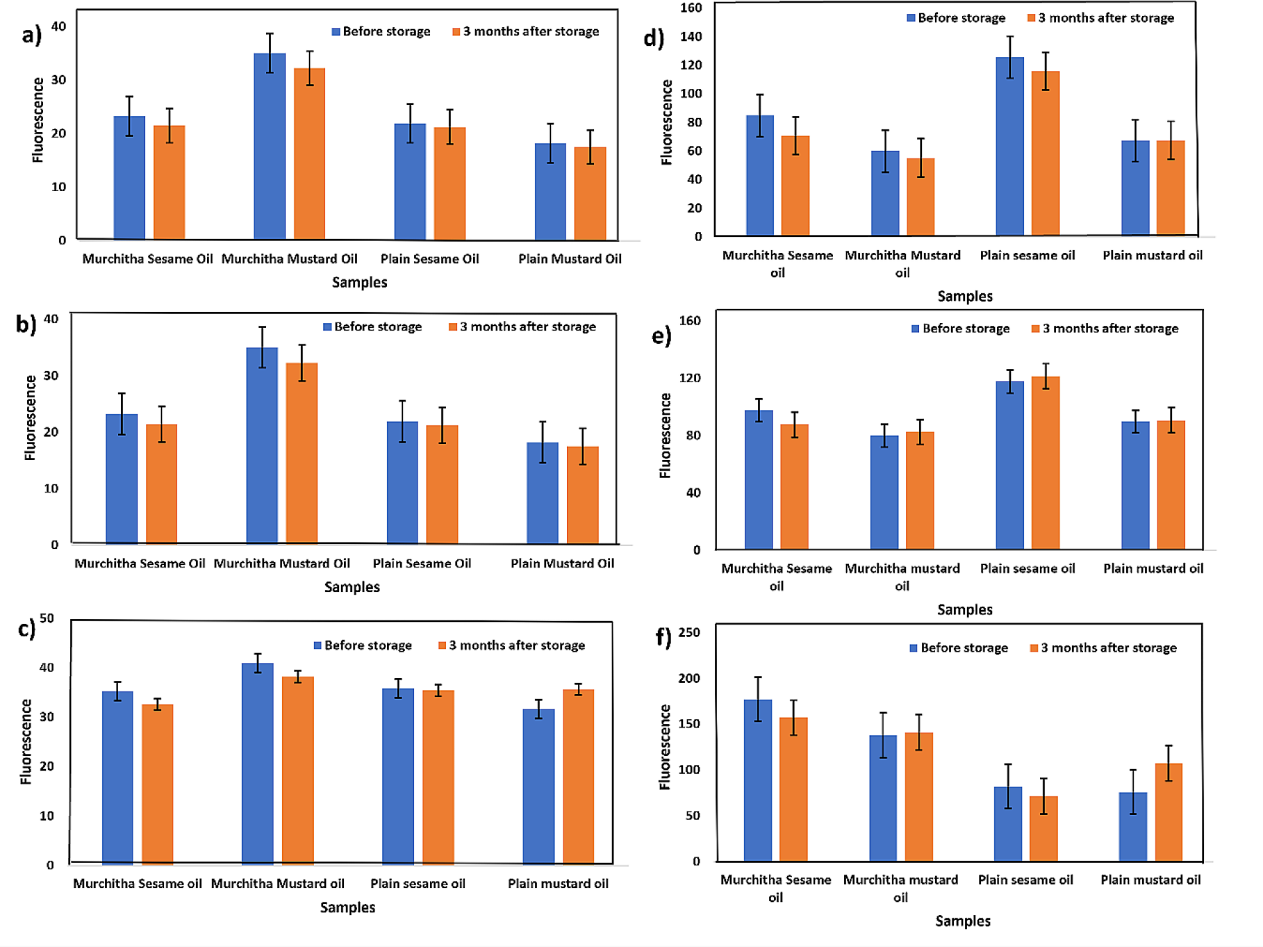
Fluorescence data **a** represents the fluorescence of oils at 300 nm excitation **b** represents the fluorescence of oils at 320 nm excitation **c** represents the fluorescence of oils at 340 nm excitation **d** represents the fluorescence of oils at 360 nm excitation **e** represents the fluorescence of oils at 380 nm excitation **f** represents the fluorescence of oils at 420 nm excitation

Fluorescence spectroscopy offers high sensitivity and selectivity for the analysis of oils, making it a powerful tool for detecting and quantifying specific compounds present in complex matrices. This technique utilizes the intrinsic fluorescence emitted by fluorophores within the oil sample when excited by light of a specific wavelength. By measuring the intensity and wavelength of the emitted fluorescence, the identification and quantification of aromatic compounds, antioxidants, and contaminants, even at low concentrations can be performed, thus justifying the addition of this analytical technique for the analysis of oil samples in this study. Additionally, fluorescence spectroscopy can provide valuable information about the structural and conformational changes occurring in oil molecules under different environmental conditions, offering insights into their stability and quality. In this study, fluorescence spectroscopy has been used to analyse the presence of fluorescing components such as chlorophyll and carotenoids in the oil samples. The fluorescence spectra at different excitation wavelengths of 300 nm to 420 nm are shown in Figs. 2 and 3. Multiple excitation wavelengths are employed for the analysis of the fluorescence properties of a sample with multiple fluorescent components. The fluorescence spectra were obtained before and after four months. The highly similar fluorescence spectra patterns are primarily due to the vitamin E group found as tocopherols with similar chemical structures. However, the distinct areas of the spectra help differentiate the oils from one another. The different peak areas in the fluorescence spectra, such as 650–730 nm, 500–600 nm and 400–500 nm, correspond to chlorophyll, vitamin E and carotenoids, respectively [34]. In plain or *amurchitha* mustard oil, chlorophyll is strongly present, whereas, in *murchitha* mustard oil, the chlorophyll peak at 673 nm is minimal. These findings indicate a possible reduction of the components contributing to oil oxidation and rancidity in *murchitha* mustard and sesame oil. We can also observe a trend similar to that of UV-Vis absorbance spectra with respect to the quality of the *amurchitha* oils over a period of time. Thus, confirming with the benefits of the *Murchana* process as discussed earlier.

### Fourier transform infrared (FTIR) spectroscopy

**Fig. 4:**
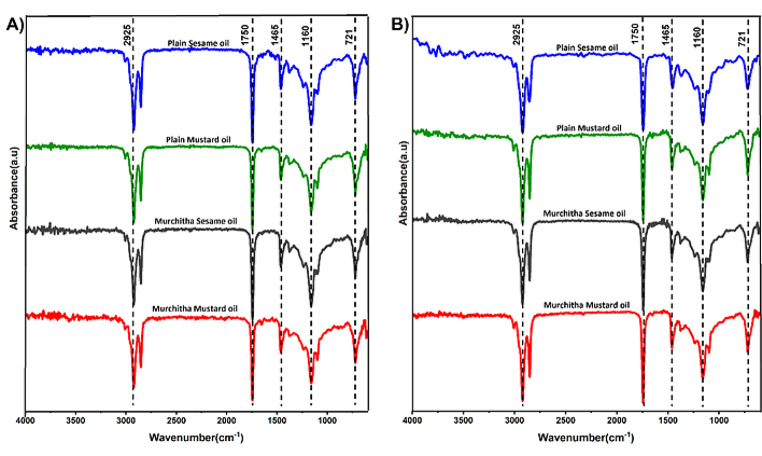
FTIR spectra of the oil samples **A** before storage and **B** 3 months after storage

FTIR spectroscopy is an analytical method for the investigation of the functional groups in a compound through the FTIR fingerprint of the compound. This offers a non-destructive and rapid method for the analysis of oils, providing valuable information about their chemical composition and structural characteristics. FTIR spectroscopy is highly sensitive to changes in molecular structure, allowing for the detection of oxidation products, contaminants, and adulterants in the oil sample. Additionally, FTIR analysis requires minimal sample preparation and can be performed on a wide range of oil types, making it suitable for both research and quality control applications in various industries [35]. FTIR spectra is used to elucidate different functional groups in the oil for each peak in the spectra represents different bonds (Table 1). The Fig. 4 represents the FTIR spectra of the oil samples before and after storage for 3 months. The peaks at 3470 cm-1 corresponds to the O-H stretch, the peaks at 2925 cm-1 and 1465 cm-1 represent the CH2 stretch. The peaks between 3000 and 2800 cm-1 indicate the C-H stretching vibration of the CH2 and CH3 aliphatic groups in the triglycerides, which are abundant in the vegetable oils. The bond around 3006 cm-1 represents the C-H stretching vibration of the cis-double bond = CH. This region is generally indicative of the adulteration of the oils [36, 37]. In the region between 1800 –1600 cm-1 there are two major peaks which correspond to the presence of high saturated fatty acids content with short carbohydrate chains, and the spectral peak around 1665 cm-1 corresponds to the C = C bond that is correlated with the presence of polyunsaturated fatty acids (PUFA) in the oil samples [38]. This is indicative of the health benefits to be derived on consumption of the oil. The peaks at 1440–1445 cm-1 indicate the vibrations due to the deformation of δ(C-H) bonds, which is used to determine the level of unsaturation. The FTIR spectra of the oils do not show a significant difference in the position of the peaks before or after storage. Thus, indicating reasonable stability of the functional groups of the oil samples under the storage conditions. This also indicates that only an FTIR analysis of oil samples might be insufficient to detect degradation of oil due to oxidation. One limitation of FTIR spectroscopy for oil analysis is its susceptibility to spectral interference from background noise and overlapping absorption bands. Vegetable oils often exhibit complex spectra due to the presence of multiple components with overlapping absorption peaks, which complicates spectral interpretation and quantification. Moreover, FTIR spectroscopy may have limited sensitivity for detecting minor components or trace contaminants in the oil samples. We can also infer that a storage period of three months maybe insufficient to correctly determine change in the quality of the oils due to photodegradation during long periods of storage. However, there is evidence suggesting the applicability of FTIR analysis for the detection of adulteration of oils [36, 39–41].

High performance liquid chromatography (HPLC) and thin layer chromatography (TLC) are analytical techniques which are employed for the separation and identification of the various components in a sample. These techniques are regularly applied in fields such as pharmaceuticals, chemistry, environmental analysis, and food science. HPLC and TLC are used as a complimentary study to supplement the findings of the present study; the results of which are included in Fig. 1 (HPLC) and Fig. 2 (TLC) in the supplementary material.

**Table 1:** Assignment of chemical bonds in the FTIR spectrum

Wavenumber (cm ^− 1^)	Assignment	Compound Class	References
1750	C = O	Esters	[23, 42]
2925	CH_2_	Alkanes	[26]
1465	C-H stretching of CH_2_ and CH_3_	Alkanes	[26]
3006	=CH stretching	Alkenes	[25]
1445	-CH_2_ bending	Methyl groups	[26]
1390	C-H bending	Aldehydes	[25]
1170	C-O stretching vibration	Methyl groups in esters	[25]
665–730	C = C	Alkenes	[22]

## Conclusions

The spectral properties of the vegetable oils have been studied using spectroscopy techniques, such as absorption, fluorescence, and FTIR. These techniques offer rapid and non-destructive means of assessing the chemical composition and structural characteristics of oils, aiding in the detection of contaminants, oxidation products, and adulterants. Their versatility allow1s for comprehensive analysis across a wide range of oil types with minimal sample preparation. This study examined the effects of the ayurvedic processing technique ‘*Murchana*’ on sesame and mustard oils. Vegetable oils can be characterized easily by studying their chlorophyll and carotenoid content. Additionally, the study illustrates that spectroscopic techniques can be used to supplement the chemical methods analysis to determine the effects of the *murchana* process on oils. The UV-Vis absorbance and fluorescence spectra of the samples were found to provide adequate data for the analysis of oil samples, thus providing an alternative method to study the constituents and nature of oil samples.

However, the methodology in this study also has its limitations. Challenges such as spectral interference from complex matrices, limited specificity for individual compound identification, and difficulties in quantitative analysis of the oils. Furthermore, these spectroscopic techniques typically provide bulk analysis without spatial resolution, potentially overlooking localized variations within the oil samples. Moreover, in the analysis of oil samples using spectroscopic techniques, the sensitivity of the method and the response time are often inversely related. Higher sensitivity, achieved through methods like longer integration times or more sensitive detectors, allows for the detection and quantification of trace-level components within the oil sample. However, this heightened sensitivity often comes at the cost of increased response time, as more data points need to be collected and analysed to achieve accurate measurements. Conversely, reducing response time, such as by using rapid scanning techniques or optimized experimental conditions, may sacrifice some sensitivity due to shorter integration times or decreased signal-to-noise ratios. Thus, there exists a trade-off between sensitivity and response time in spectroscopic analysis of oil samples. Addressing these limitations necessitates careful consideration of experimental design, method optimization, and complementary analytical approaches to ensure the reliability and interpretability of the research findings, necessitating further studies to ascertain the beneficial effects of the *murchana* process on vegetable oils. Further investigations could be conducted on the oil samples to ascertain the impact of prolonged storage duration, specifically by extending the storage period as well.

## Electronic supplementary material

Below is the link to the electronic supplementary material.

Supplementary Material 1
