# Supplementary material for: Spectroscopic evaluation of sesame and mustard oils treated with Murchana method
Source: Lasers Med Sci. 2024 Apr 11;39(1):99. doi: 10.1007/s10103-024-04050-x (PMC11008066; doi:10.1007/s10103-024-04050-x)
Supplement: Supplementary file 1 — Supplementary Material 1 [file 10103_2024_4050_MOESM1_ESM.docx]

**Spectroscopic Analysis of Sesame and Mustard Oils Prepared Using the *Murchana* Method**

**Supplementary Material:**

1. High Performance Liquid Chromatography (HPLC):

HPLC was used to separate components of the oil samples (*Murchitha* Sesame, *Murchitha* Mustard, Plain Sesame, and Plain Mustard). α – tocopherol (Sigma Aldrich) was used as a standard for the analysis. The HPLC procedure was performed in Shimadzu Ultra Fluorescence Liquid Chromatography (UFLC). The detector was set at excitation and emission wavelength of 290 nm and 330 nm respectively. Methanol (50%) and Acetonitrile (50%) were used as mobile phases at a flow rate of 1 ml/min.C18 column of 250 mm ×4.6 mm was used in the estimation, and the temperature was set to 40 °C. The injection volume of the sample was 5 µl. The oil samples were prepared in isopropanol in 1:10 ratio. The standard α- tocopherol was prepared in ethanol and later dissolved in isopropanol in a 1:100 ratio [1]. Figure 1 represents the analysis of the sample oils by HPLC.


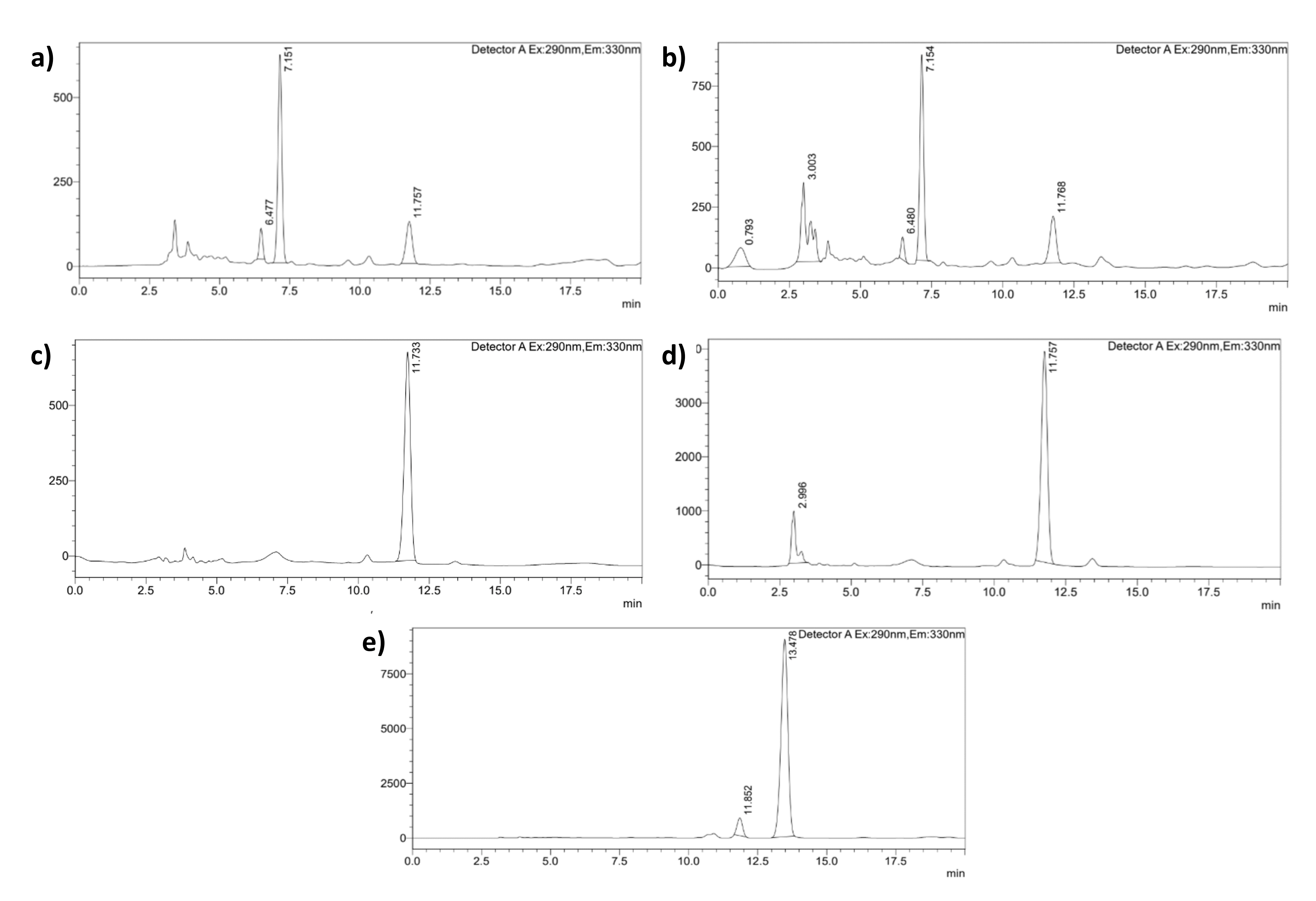


Figure 1: HPLC analysis of a) Plain Sesame oil b) *Murchitha* Sesame oil c) Plain Mustard oil d) *Murchitha* Mustard oil e) Standard α – tocopherol

1. Thin Layer Chromatography (TLC):

TLC was performed on the samples. The procedure was performed using Macherey- Nagel, DC- Fertigfolien Alugram Xtra SIL G/UV 254 (20× 20 cm). CAMAG Automatic TLC Sampler 4 was used to visualize the TLC plate at wavelength 254 nm, 366 nm. Twin Trough Chamber was also used to run the TLC. The standard α- tocopherol was prepared in ethanol and later dissolved in isopropanol in a 1:100 ratio by dissolving 10 µl of α- tocopherol in 1000 µl of isopropanol. The oil samples were prepared in isopropanol in a 1:10 ratio by dissolving 10 µl of the oil sample in 100 µl of isopropanol. 2 µl of standard and oil samples were applied on the TLC plate (20× 20 cm). Acetronitrile and methanol (1:1) were used as the mobile phase. After the application of Samples, the TLC plate was immersed in the TLC trough containing the mobile phase. The samples were visualized at wavelengths 254 nm and 366 nm. Figure 2 represents the TLC plate. The samples corresponding to the spots on the TLC plate, and the retention factor is represented in table 1.


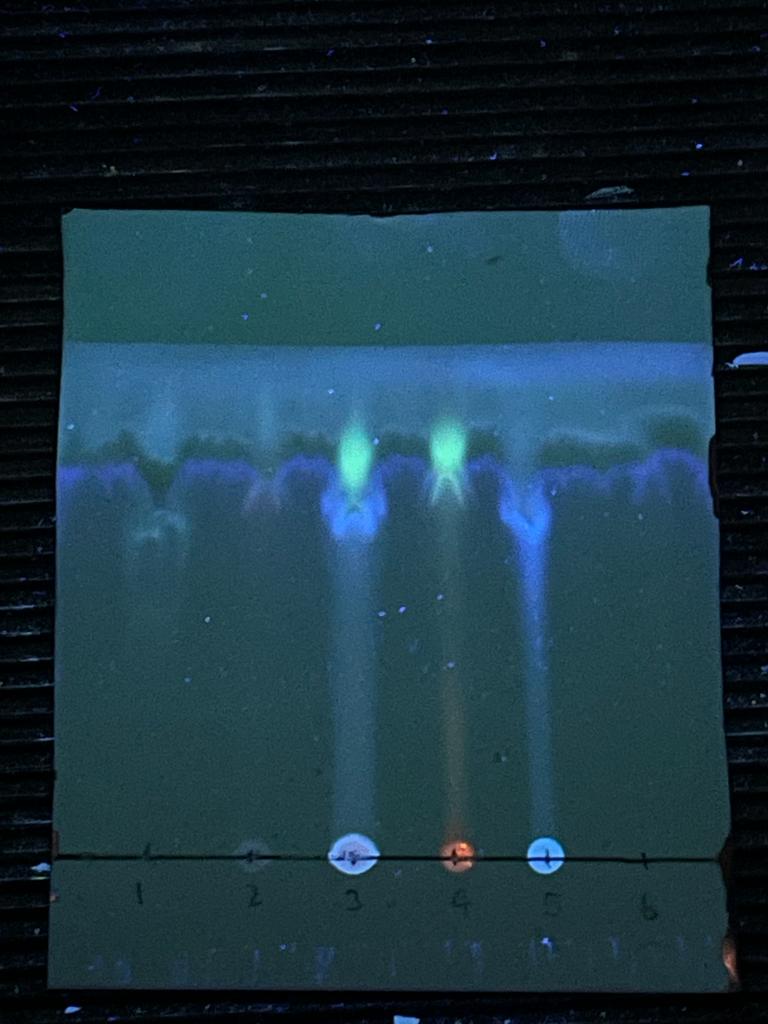


Figure 2: TLC plate visualised at 366 nm.

| **Spot** | **Sample name** | **Rf** |
| --- | --- | --- |
| 1 | Standard α - tocopherol | 0.70 |
| 2 | Plain mustard oil | - |
| 3 | *Murchitha* sesame oil | 0.70 (Spot 1) |
| 3 | *Murchitha* sesame oil | 0.78 (Spot 2) |
| 4 | *Murchitha* mustard oil | 0.78 |
| 5 | Plain sesame oil | 0.70 |

**References:**

1. Bakre SM, Gadmale DK, Toche RB, Gaikwad VB. Rapid determination of alpha tocopherol in olive oil adulterated with sunflower oil by reversed phase high-performance liquid chromatography. Journal of Food Science and Technology. 2015;52:3093-3098.
2. Kumar UP, Shree AR. A rapid technique for detection and quantification of mineral oil in vegetable oils used as vehicles in ayurvedic formulations. International Journal of Herbal Medicine. 2014;2:20-22.
